# LncRNA-Based Classification of Triple Negative Breast Cancer Revealed Inherent Tumor Heterogeneity and Vulnerabilities

**DOI:** 10.3390/ncrna8040044

**Published:** 2022-06-21

**Authors:** Radhakrishnan Vishnubalaji, Ramesh Elango, Nehad M. Alajez

**Affiliations:** 1 Translational Cancer and Immunity Center (TCIC), Qatar Biomedical Research Institute (QBRI), Hamad Bin Khalifa University (HBKU), Qatar Foundation (QF), Doha P.O. Box 34110, Qatar; vbradhakrishnan@hbku.edu.qa (R.V.); relango@hbku.edu.qa (R.E.); 2 College of Health & Life Sciences, Hamad Bin Khalifa University (HBKU), Qatar Foundation (QF), Doha P.O. Box 34110, Qatar

**Keywords:** lncRNA, triple negative breast cancer, classification, CRISPR, Cas9

## Abstract

Triple negative breast cancer (TNBC) represents a diverse group of cancers based on their gene expression profiles. While the current mRNA-based classification of TNBC has contributed to our understanding of the heterogeneity of this disease, whether such heterogeneity can be resolved employing a long noncoding RNA (lncRNA) transcriptome has not been established thus far. Herein, we used iterative clustering and guide-gene selection (ICGS) and uniform manifold approximation and projection (UMAP) dimensionality reduction analysis on a large cohort of TNBC transcriptomic data (TNBC = 360, normal = 88) and classified TNBC into four main clusters: LINC00511-enriched, LINC00393-enriched, FIRRE-enriched, and normal tissue-like. Delving into associated gene expression profiles revealed remarkable differences in canonical, casual, upstream, and functional categories among different lncRNA-derived TNBC clusters, suggesting functional consequences for altered lncRNA expression. Correlation and survival analysis comparing mRNA- and lncRNA-based clustering revealed similarities and differences between the two classification approaches. To provide insight into the potential role of the identified lncRNAs in TNBC biology, CRISPR-Cas9 mediated LINC00511 promoter deletion reduced colony formation and enhanced the sensitivity of TNBC cells to paclitaxel, suggesting a role for LINC00511 in conferring tumorigenicity and resistance to therapy. Our data revealed a novel lncRNA-based classification of TNBC and suggested their potential utilization as disease biomarkers and therapeutic targets.

## 1. Introduction

Triple negative breast cancers (TNBCs) are invasive mammary carcinomas (otherwise, invasive ductal carcinomas) with a more aggressive nature compared to other breast cancer (BC) subtypes. Patients with TNBC have a higher rate of relapse, with relatively poorer outcomes for stage-compared other breast cancer subtypes expressing ER/PR and HER2 molecular targets [1,2]. TNBCs show a higher rate of metastases to the brain, liver, and lung and lower rates to the bone than those reported for other subtypes of BC [3]. The intrinsic classification of BC performed by the PAM 50 (Prediction Analysis of Microarray) gene set added prognostic and predictive value to the clinical staging and histological grading [4]. Lehmann et al. defined six TNBC subtypes based on the PAM gene set and ontologies: immunomodulatory subgroups (IM); luminal androgen receptor (LAR); two basal-like (BL1 and BL2) and mesenchymal (M) and mesenchymal stem-like (MSL)) from cluster analysis, each exhibiting a unique biology, in addition to one unstable group (UNS), which clustered away from these six subtypes [5].

In addition to the above-mentioned mRNA-based TNBC classification, many studies have shown the heterogeneity of BC and its impact on cellular functions, especially genetic and non-genetic alterations [6]. Nonetheless, we recently classified TNBC into seven clusters based on their mRNA transcriptome, each exhibiting a unique molecular signature [7]. The use of genomics and transcriptomic approaches is currently gaining momentum to better understand TNBC heterogeneity and its implications in tumor response to neoadjuvant chemotherapy [8,9].

While the bulk of research on the molecular classification of BC has focused on protein-coding mRNAs, the use of lncRNA-based classification is gaining momentum, given their tissue-specific expression patterns [10]. In fact, a comprehensive classification of breast cancer subtypes was recently performed employing their lncRNA transcriptional portraits [11]. However, currently there is a lack of knowledge on the utilization of lncRNA-based classification to reveal the heterogeneity of TNBC and their clinical implications. In the current study, we employed integrated transcriptomic, computational, and genome editing approaches and classified TNBC into four major clusters based on their lncRNA transcriptomes, unraveling their functional heterogeneity and correlation with clinical outcomes. Here, we report a novel approach to classifying TNBC based on their lncRNA transcriptome and suggest potential implications in the clinical management of TNBC.

## 2. Results

### 2.1. Differentially Expressed LncRNAs in TNBC Compared to Normal Breast Tissue (NT)

**Figure 1:**
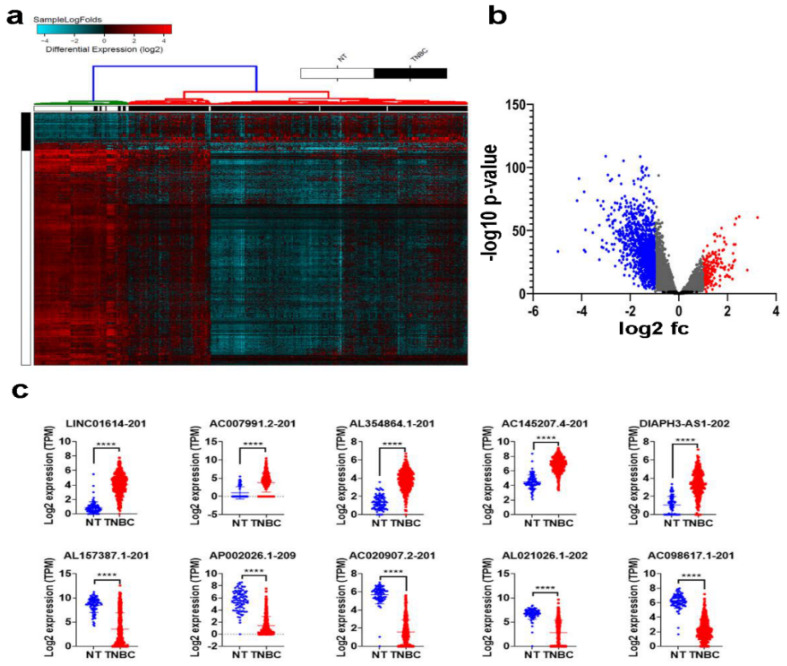
Differentially expressed lncRNAs in TNBC compared to normal breast tissue (NT). RNA-Seq data were aligned to the human geocode release v33, and the abundance of each lncRNA transcript was quantified. (**a**) Hierarchical clustering of TNBC (n = 360) compared to NT (n = 88). Each column represents one sample, and each row represents the lncRNA transcript. The expression level of each transcript (log2) is depicted according to the color scale. (**b**) Volcano plot depicting upregulated (red) and downregulated (blue) lncRNAs in TNBC compared to NT. (**c**) Scatter plot depicting the expression of the top 5 upregulated (upper panel) and downregulated (lower panel) lncRNA transcripts in TNBC compared to NT. **** *p* < 0.00001.

To provide a global view of the alternations in lncRNA expression associated with TNBC, RNA-Seq data from 360 TNBC patients and 88 normals were aligned to the human geocode release v33, and the abundance of a total of 48,351 lncRNA transcripts was quantified. Differential analysis identified 187 upregulated and 1386 downregulated lncRNA transcripts in TNBC compared to adjacent normal tissue (NT), 2.0 fc, *p* (Adj) < 0.05, Figure 1a) and Supplementary Table S1. The volcano plot depicting the differentially expressed lncRNAs in TNBC vs. NT is shown in Figure 1b. The expression of the top 5 upregulated and top 5 downregulated lncRNA transcripts is illustrated in Figure 1c, with LINC01614-201 being top upregulated and AL157387.1-201 being top downregulated lncRNA transcript in TNBC. 

### 2.2. Molecular Heterogeneity of TNBC Employing the LncRNA Transcriptome

**Figure 2:**
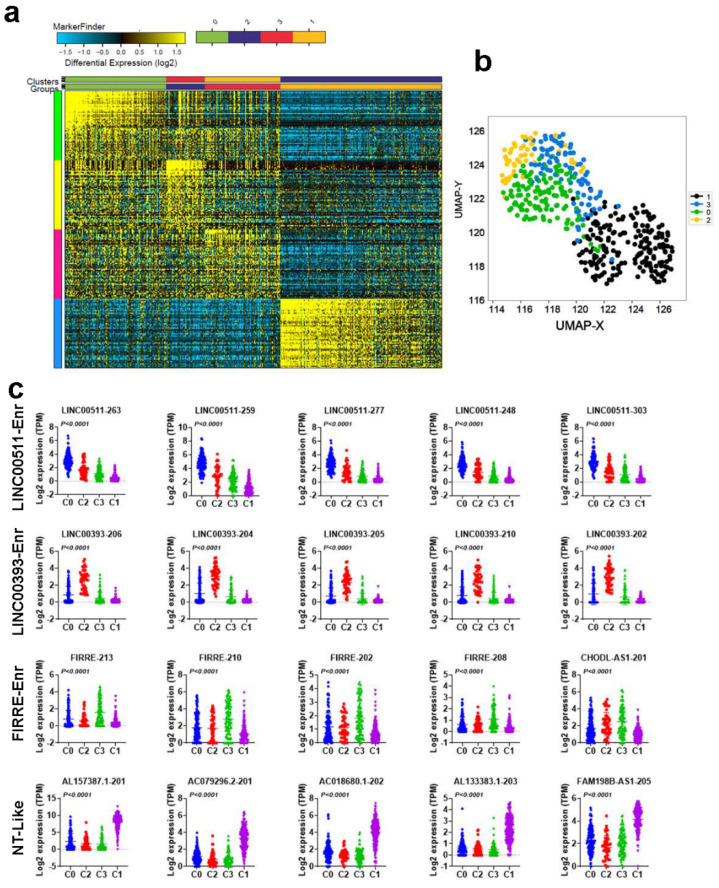
Molecular heterogeneity of TNBC employing lncRNA transcriptome. (**a**) Iterative clustering and guide-gene selection (ICGS) classification of TNBC and NT based on their lncRNA transcriptome, revealing four distinct clusters. (**b**) Uniform manifold approximation and projection (UMAP) dimensionality reduction analysis of TNBC and NT based on their lncRNA expression. (**c**) Scatter plot depicting the expression of the top 5 enriched lncRNA transcripts in each cluster (C0, C2, C3, and C1) in the same cohort.

We subsequently sought to classify TNBC (TNBC = 360 and normal = 88) based on their lncRNA transcriptome employing the ICGS algorithm. Using this approach, we were able to classify TNBC and NT into four main clusters (C0, C2, C3, and C1), with each cluster having a distinctive lncRNA expression profile (Figure 2a) and Supplementary Table S2. Based on the enrichment of selected lncRNA transcripts, the four clusters were designated LINC00511-enriched (551-Enr), LINC00393-enriched (393-Enr), FIRRE-enriched (FIRRE-Enr), and normal tissue-like (NT-like), respectively. The NT-like cluster clustered with the NT. A similar pattern was also seen when utilizing the UMAP dimensionality reduction analysis (Figure 2b). Expression of the top 5 lncRNA transcripts in each cluster (C0, C2, C3, and C1) in the same cohort is shown in Figure 2c.

### 2.3. Functional Heterogeneity of TNBC Employing LncRNA-Based Clustering

**Figure 3:**
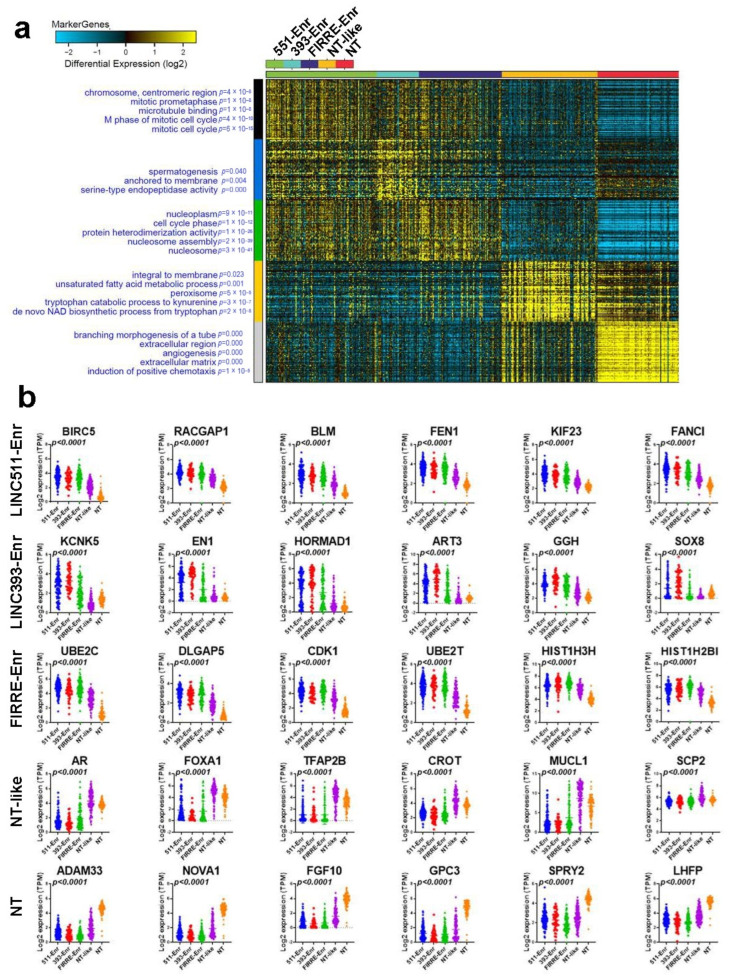
Marker finder analysis depicting gene and functional categories associated with the four lncRNA-based clusters. (**a**) Marker finder heatmap illustrating the expression and associated functional categories (left) in each cluster (LINC00551-Enr, LINC00393-Enr, FIRRE-Enr, NT-like, and NT). (**b**) Scatter plot depicting the expression of the top enriched genes in each cluster (LINC00551-Enr, LINC00393-Enr, FIRRE-Enr, NT-like, and NT). The corresponding *p* values are indicated in each plot. 551-Enr, LINC00511-enriched; 393-Enr, LINC00393-enriched; FIRRE-Enr, FIRRE-enriched; NT-like, normal tissue-like; NT, normal tissue.

We subsequently divided the cohort into four groups based on their lncRNA classification and identified their corresponding protein-coding transcriptomes. Employing the marker finder algorithm, we observed significant differences in the enriched gene ontology (GO) annotations associated with each TNBC cluster (Figure 3a) and Supplementary Table S3.

All three, but the NT-like, clusters were enriched in cell proliferation-associated functions. In particular, the LINC00511-Enr cluster was more enriched in functional annotations, indicating cell cycle and mitotic activity, while the LINC00393-Enr cluster was more enriched in serine-type endopeptidase activity. The FIRRE-Enr was associated with functional categories indicative of nucleosome assembly and cell cycle, while the NT-like cluster was as enriched in de novo NAD biosynthesis and the unsaturated fatty acid metabolic process. Normal breast tissue was enriched in chemotaxis, angiogenesis, and extracellular matrix functional categories. The expression of the top-enriched genes in each cluster is shown in Figure 3b. The LINC00511-Enr cluster was enriched in BIRC5, RACGAP1, BLM, FEN1, KIF23, and FANCI; the LINC00393-Enr cluster was enriched in KCNK5, EN1, HORMAD1, ART3, GGH, and SOX8; the FIRRE-Enr cluster was enriched in UBE2C, DLGAP5, CDK1, UBE2T, HIST1H3H, and HIST1H2BI; the NT-like cluster was enriched in AR, FOXA1, TFAP2B, CROT, MUCL1, and SCP2; while the NT cluster was enriched in ADAM33, NOVA1, FGF10, GPC3, SPRY2, and LHFP.

### 2.4. Upstream Regulator and Functional Annotation Enrichment in LncRNA-Derived TNBC Clusters

**Figure 4:**
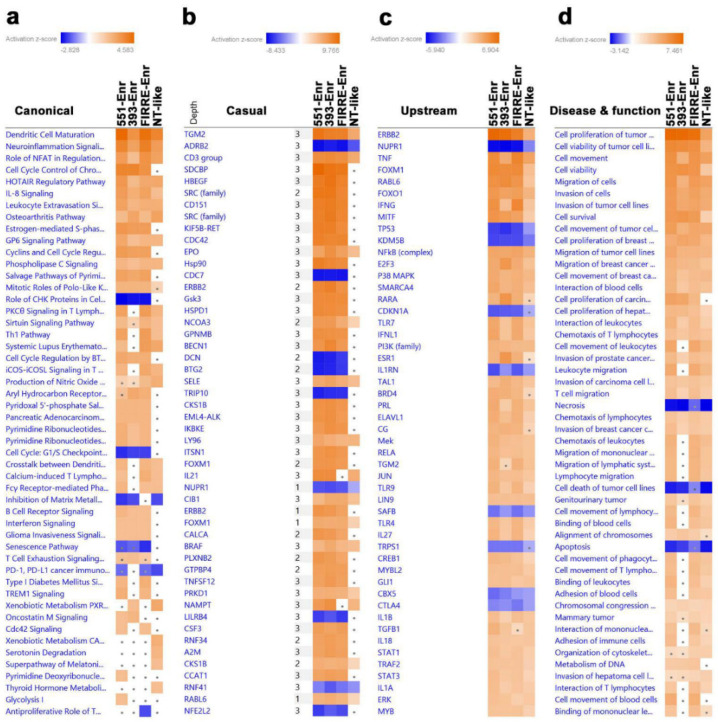
Upstream regulator and functional annotation enrichment in lncRNA-derived TNBC clusters. Upregulated genes from each lncRNA-derived TNBC cluster compared to NT were subjected to comparative casual (**a**), canonical (**b**), upstream (**c**), and disease and function (**d**) analysis using IPA illustrating the top affected categories. Color intensity corresponds to the activation Z-score. 551-Enr, LINC00511-enriched; 393-Enr, LINC00393-enriched; FIRRE-Enr, FIRRE-enriched; NT-like, normal tissue-like; NT, normal tissue.

To gain more insight into the enriched pathways and signaling networks in each lncRNA cluster, upregulated genes in each TNBC cluster compared to NT were imported into IPA and subjected to canonical, casual, upstream, and disease and function analyses. The data presented in Figure 4a suggest activation of immune functions canonical (i.e., Dendritic cell Maturation, PKC*θ*, and Th1) in the LINC00511-Enr and FIRRE-Enr clusters. The LINC00393-Enr and NT-like clusters were depleted from the majority of immune-related canonical pathways.

Similar functional differences were also observed with casual network analysis (Figure 4b). TNF and IFNG upstream regulators were highly enriched in the LINC00511-Enr and FIRRE-Enr clusters, while E2F3, RARA, and ESR1 were enriched in the LINC00393-Enr cluster (Figure 4c). The LINC00511-Enr and FIRRE-Enr clusters were more enriched in leukocyte movement and migration, while the cell proliferation and survival of tumor cells were similar for the LINC00511-Enr, FIRRE-Enr, and LINC00393-Enr clusters (Figure 4d). These data support the existence of functional differences in the identified TNBC clusters.

**Figure 5:**
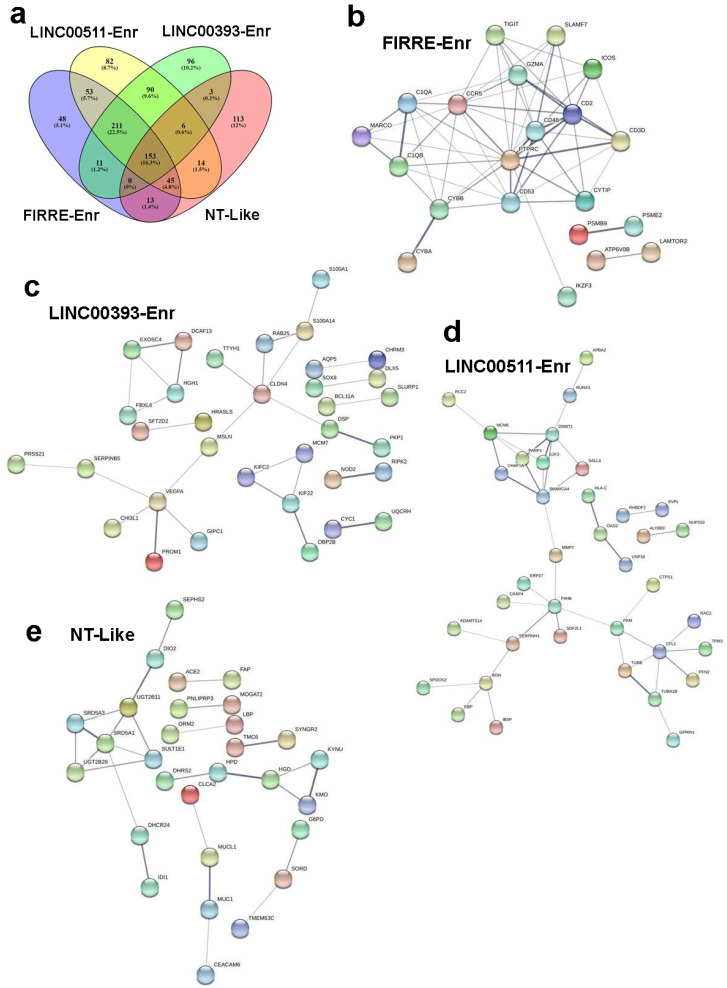
Protein–protein interaction (PPI) network analysis on upregulated genes in the indicated lncRNA-based h TNBC clusters. (**a**) Venn diagram depicting the commonality and uniquely upregulated genes in each lncRNA-derived cluster. STRING PPI network of uniquely upregulated genes in FIRRE-Enr (**b**), LINC00511-Enr (**c**), LINC00393-Enr (**d**), and NT-like (**e**) clusters. 551-Enr, LINC00511-enriched; 393-Enr, LINC00393-enriched; FIRRE-Enr, FIRRE-enriched; NT-like, normal tissue-like; NT, normal tissue.

To gain deeper insight into PPI and signaling networks that underline the observed functional differences, upregulated genes in each lncRNA-based cluster compared to NT were subjected to PPI network analysis using the Search Tool for the Retrieval of Interacting Genes/Proteins (STRING). The FIRRE-Enr cluster network exhibited 429 nodes, 9277 edges with an average node degree of 43.2 and an average local clustering coefficient of 0.568 and PPI enrichment *p*-value of <1.0^−16^ (Supplementary Figure S1). The highest enrichment based on GO analysis was for cell cycle progression (Supplementary Table S4). The FIRRE-Enr cluster was characterized by the presence of immune infiltration, including the presence of CTLA4 and SLAMF7. The LINC00511-Enr network exhibited 539 nodes and 11,067 edges, with an average node degree of 41.1, average local clustering coefficient of 0.531, and PPI enrichment *p*-value of <1.0 × 10^−16^ (Supplementary Figure S2). This cluster was enriched in mitotic DNA replication and the presence of immune infiltration, including CTLA4, but lacked SLAMF7 (Supplementary Table S5). The LINC00393-Enr cluster exhibited 492 nodes, 9799 edges, an average node degree of 39.8, and an average local clustering coefficient of 0.538. The network exhibited a PPI enrichment *p*-value of <1.0 × 10^−16^ (Supplementary Figure S3). This cluster revealed cell cycle activation as the main enriched GO category (Supplementary Table S6). The normal-like cluster had 234 nodes, 1428 edges, an average node degree of 12.2, an average local clustering coefficient of 0.569, and a PPI enrichment *p*-value of <1.0 × 10^−16^ (Supplementary Figure S4). This cluster exhibited significant immune infiltration but a lack of CTLA4 and SLAMF7 expression (Supplementary Table S7). To delineate functional differences among lncRNA-based clusters, we focused on the set of genes that were uniquely upregulated in each cluster and identified 48 genes enriched in FIRRE-enr, 82 genes enriched in LINC00511-Enr, 96 genes enriched in LINC00393-Enr, and 113 genes enriched in the NT-like cluster (Figure 5a). PPI analysis of those gene sets revealed immune system processes as the hallmark of the FIRRE-Enr cluster, serine protease as the hallmark of the LINC00393-Enr cluster, extracellular space as the hallmark of the LINC-00511-Enr cluster, and small molecule and lipid metabolic as the main feature of the NT-like cluster (Figure 5b–e). The immune signature unique to FIRRE-Enr with the highest score was for lymphocyte activation (CD3D, LY6D, IKZF3, SLAMF7, CD2, PTPRC) and regulation of lymphocyte activation (THOC1, ICOS, IKZF3, CD2, PTPRC, TIGIT). Taken together, our data revealed enrichment in the cell cycle and immune functions as the predominant functional categories in different lncRNA-based clusters.

### 2.5. Survival Analysis of TNBC Patients Employing LncRNA-Based Classification

To put our lncRNA-based clustering and functional analysis into context, the TNBC cohort (n = 360) was subjected to Kaplan–Meier survival analysis as a function of their corresponding lncRNA-based cluster. The LINC00393-Enr cluster correlated with the worst RFS compared to the other clusters (Supplementary Figure S5a). Interestingly, our lncRNA-based classification predicted the group with the worst clinical outcome; however, mRNA-based classification predicted the group of patients with a better outcome (Supplementary Figure S5b), suggesting the usefulness of our lncRNA-based classification in predicting patients with a higher probability of RFS. Correlative analysis of lncRNA-base and mRNA-based classifications revealed the FIRRE-Enr cluster to overlap with BLIS and IM, the LINC00393-Enr cluster to overlap with BLIS, the LINC00511-Enr cluster to overlap with BLIS, and to a lesser extent with IM, while the NT-like cluster to overlap with LAR and to lesser extent with MES, which would be concordant with the functional and PPI analysis (Supplementary Figure S5c).

### 2.6. CRISPR-Cas9-Mediated LINC00511 Suppression Abrogated TNBC CFU Formation and Enhanced Their Sensitivity to Paclitaxel

**Figure 6:**
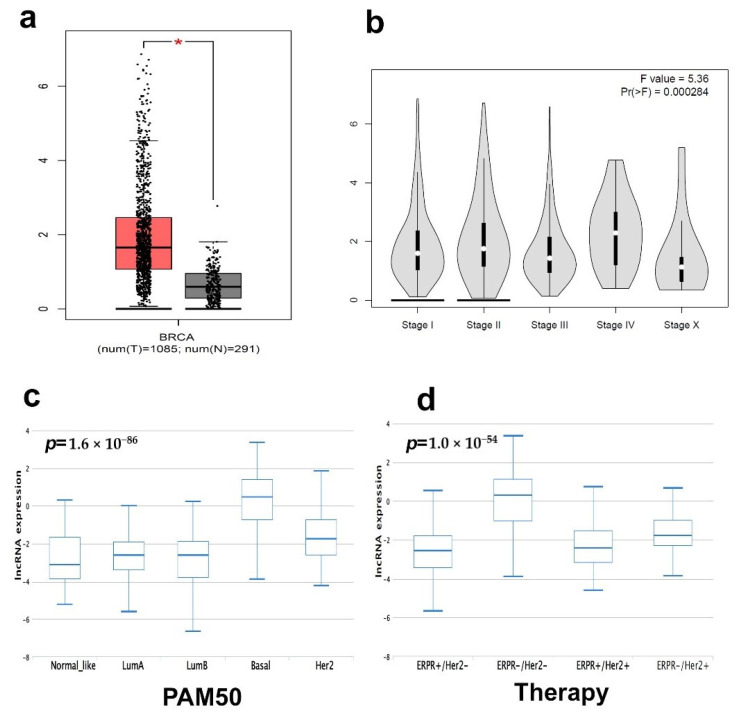
Expression of LINC00511 and its correlation with pathological stage in BC. (**a**) Expression of LINC00511 in BC (n = 1085) compared to normal breast tissue (n = 291) from the BRCA TCGA cohort. (**b**) Stage plot demonstrating LINC00511 expression as a function of pathological stage in the BRCA TCGA breast cancer cohort (n = 1085). Expression on LINC00511 as a function of PAM50 classification (**c**) or as a function of therapy (**d**) based on the TANRIC database. * *p* < 0.05.

To extend our findings into an additional BC cohort, the expression of LINC00511 was assessed in a large BC cohort (n = 1085) and normal controls (n = 291) from the TCGA BRCA dataset, which revealed a high expression of LINC00511 in BC compared to normal (Figure 6a). Interestingly, the expression of LINC00511 correlated with the advanced BC pathological stage (Figure 6b), suggesting that LINC00511 is a potential therapeutic target for BC. In accordance with our data, the expression of LINC00511 was highest in the basal (Figure 6c) and in patients receiving ERPR-/HER2-therapy (Figure 6d).

**Figure 7:**
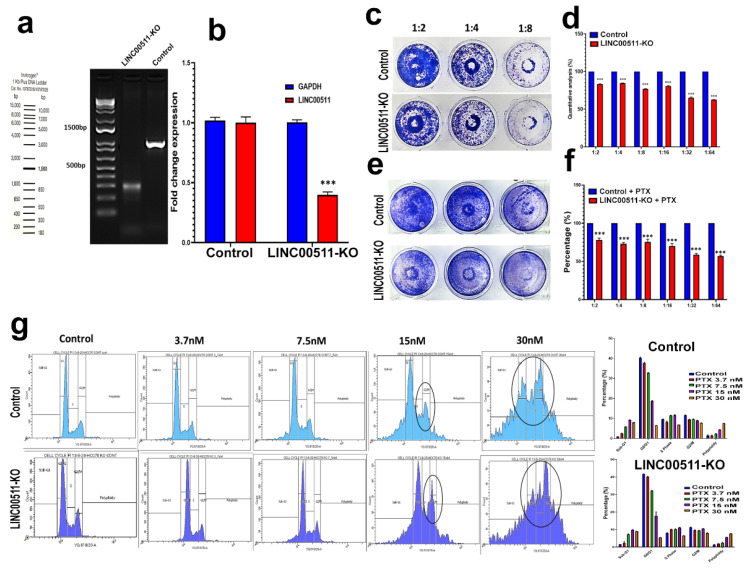
Effect of CRISPR-Cas9 mediated LINC00511 promoter deletion on HCC70 colony formation and paclitaxel sensitivity. (**a**,**b**) Genomic deletion of the LINC00511 promoter using CRISPR-Cas9 in the HCC70 TNBC model. qRT-PCR for LINC00511 expression in HCC70 WT and LINC00511-KO cell models. Data are presented as mean ± SD, n = 3. *** *p* < 0.0005. Clonogenic assay for HCC70 parental and LINC00511-KO cells, in the absence (**c**,**d**) or the presence (**e**,**f**) of indicated concentrations of Paclitaxel. Data are presented as mean ± SD, n = 4. *** *p* < 0.0005. (**g**) Cell cycle analysis for HCC70 parental and LINC00511-KO cells in the absence or presence of different concentrations of Paclitaxel.

We subsequently employed CRISPR-Cas9 technology to understand the role of the selected lncRNA, LINC00511, in TNBC biology and response to neoadjuvant chemotherapy, given its abundant expression in TNBC. CRISPR-Cas9 was used to delete the promoter region of LINC00511 in the HCC70 TNBC model using our previously described system [12]. Genomic PCR of the LINC00511 promoter region revealed successful deletion of ~700 from the LINC00511 promoter in the KO model but not the parental control (Figure 7a). qRT-PCR confirmed downregulation of LINC00511 expression in the LINC00511-KO model compared to the WT, while GAPDH was used as loading control (Figure 7b). A colony-forming unit (CFU) assay was performed to look at the perturbational effects of LINC0051 depletion, which revealed a significant reduction in cell proliferation in the LINC00511-KO model compared to parental WT as a single treatment modality or in combination with paclitaxel (Figure 7c–f). Concordantly, cell cycle analysis with different concentrations of Paclitaxel shows enhanced effects of LINC0051 depletion when combined with 15 and 30 nM paclitaxel, leading to cell arrest in G2/M phase and an increased number of apoptotic cells (Figure 7g). Similar results were also observed when using the MDA-MB-157 TNBC model, thus corroborating a role for LINC00511 in modulating TNBC proliferation and sensitivity to Paclitaxel (Supplementary Figure S6).

**Figure 8:**
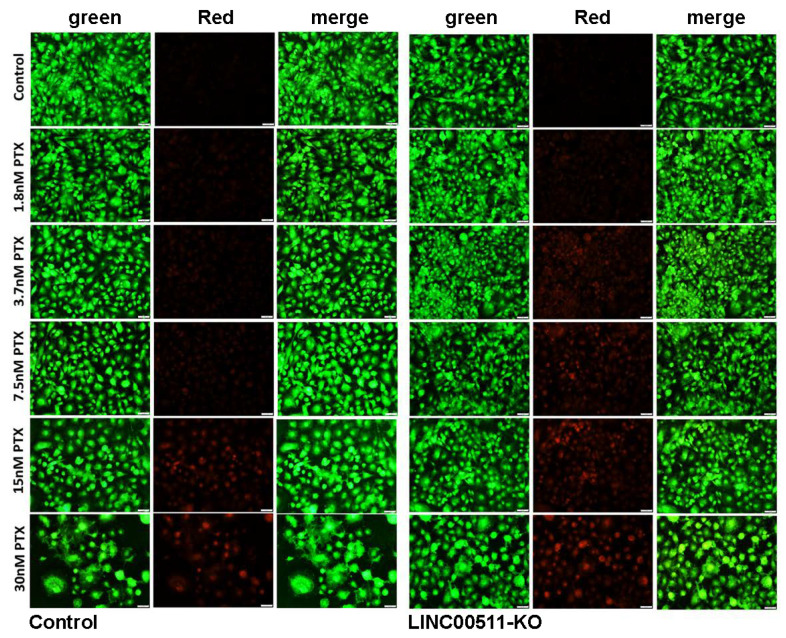
Viability staining for LINC00511-KO cells in the absence or presence of Paclitaxel. Representative fluorescence images of HCC70 parental and LINC00511-KO cells (±different concentration (1.8–30 nM) Paclitaxel). The cells were stained with acridine orange/ethidium bromide to detect live and dead cells.

AO/EtBr viability staining also confirmed the induction of cell death in LINC00511-KO cells when combined with as low as 3.7 nM of paclitaxel (Figure 8).

## 3. Discussion

TNBC represents a major clinical challenge due to its inherent tumor heterogeneity and variable response to anti-cancer therapies [13]. In the context of NAC therapy, mounting evidence suggests that pre-existing tumor heterogeneity and clonal evolution are the main mechanisms driving resistance [8]. Recent advances in genomic research and subsequent mRNA-based classification of TNBC employing mRNA transcriptome have contributed to our understanding of the heterogeneity of this disease [5]. However, such a classification does not provide a full explanation of TNBC heterogeneity and variable response to therapy. We recently employed lncRNA transcriptomes to classify breast cancer, taking into consideration their molecular subtypes, which revealed distinct lncRNA profiles associated with each molecular subtype [11,14]. However, whether lncRNA transcriptome can be used to classify TNBC and better resolve tumor heterogeneity has not yet been explored. Herein, we initially observed that the majority of lncRNA transcripts were downregulated in TNBC compared to NT in a large cohort of TNBC (n = 380) compared to normal breast tissue (n = 88), suggesting global transcription suppression. Interestingly, we showed that TNBC can be classified based on their lncRNA transcriptional portrait into four major clusters, namely LINC00511-Enr, LINC00393-Enr, FIRRE-Enr, and NT-like, each exhibiting a unique lncRNA signature. Our lncRNA-based clustering revealed remarkable functional differences among the four lncRNA-based clusters. The expression of selected lncRNA transcripts in each TNBC cluster might reflect the activated cellular programs and signaling networks in each cluster [15]. Interestingly, lncRNA-based stratification revealed a trend in predicting patients with shorter RFS, suggesting its potential utilization in predicting patients’ outcomes. While LINC00393-Enr showed the worst clinical outcome in TNBC, this lncRNA was not expressed in any of the TNBC models in vitro, suggesting that this could be expressed by the tumor microenvironment; hence, no functional studies were carried out on this lncRNA. VEGFA is a unique gene expressed in the LINC00393-Enr cluster, which implies active angiogenesis. Additionally, we observed the expression of PROM1 (CD133) in the LINC00393-Enr cluster. PROM1 is a stem cell marker that has been linked to tumor-initiating cells and worse clinical outcomes in several human cancers [16,17]. Therefore, the expression of VEGFA and PROM1 might explain, in part, the observed poor clinical outcomes. The FIRRE-Enr cluster had unique immune signatures, including CD2, CD53, CD48, and CD3D, in addition to TIGIT and SLAMF7, which are involved in immune regulation. This would be concordant with our correlation analysis, where we observed the FIRRE-Enr cluster to overlap mostly with the BLIS and IM TNBC clusters. Wang et al. reported FIRRE in colorectal cancer (CRC) to interact with polypyrimidine tract-binding protein (PTBP1) and to promoter tumorgenicity via stabilization of BECN1 mRNA and facilitating autophagy [18]. In diffuse large cell leukemia, FIREE was found to be regulated by MYC and to induce tumorigenesis via activation of the Wnt/β-catenin pathway [19]. Aroel for FIRRE has also been reported in hepatocellular carcinoma through the regulation of PFKFB4 expression [20]. In BC, FIRRE is among the lncRNAs identified to be associated with brain metastasis [21]. While published data have implicated FIRRE as an oncogenic lncRNA in several cancer types, the role played by FIRRE in TNBC remains to be investigated mechanistically.

The existence of functional differences among lncRNA-derived TNBC clusters in canonical, casual, upstream, and disease and functional categories signifies the importance of our findings. Our data revealed low immune enrichment in the LINC00393-Enr cluster, while the LINC00511-Enr cluster was found to exhibit activation of the majority of the canonical pathways, including the glycolysis and pyrimidine deoxyribonucleic acid pathways. In one study, epigenetically dysregulated LINC00393 at the enhancer element was found to be associated with BC prognosis, where patients with higher expression of LINC00393 exhibited shorter survival times in the basal subtype [22].

Increasing evidence has shown the functional involvement of specific lncRNAs in cellular alteration, oncogenesis, metastasis, and therapy response [23]. However, few lncRNAs have been reported to be involved in breast cancer tumorigenicity [24]. Recently, as a game changer with more accurate and multipotential genome-editing technology, the CRISPR-Cas9 system has revolutionized the field of cancer research [25]. We recently employed CRISPR-Cas9 technology and reported the role of MALAT1 in TNBC response to doxorubicin and paclitaxel resistance [12].

CRISPR-Cas9-mediated LINC00511 promoter deletion in HCC70 cells reduced colony formation potential, arresting the cells in G2/M phase and increased their sensitivity to paclitaxel, suggesting a role for LINC00511 in promoting resistance, which was consistent with data using the MDA-MB-157 model. Concordant with our findings, LINC00511 was shown to accelerate the G1/S shift and prevent apoptosis in ER-negative breast cancer [26], and manipulation of LINC00511 expression confirmed a role in tumorigenesis and stemness through miR-185-3p/E2F1/Nanog axis in breast cancer [27]. Similarly, silencing of LINC00511 inhibited TGF-β1-induced migration and invasion, down-regulated MMP expression (MMP2, MMP9, MMP12) and epithelial-mesenchymal transition (N-cadherin, Vimentin, snail, and ZEB2), and enhanced E-cadherin in TGF-β1 treated non-small lung cancer cells [28]. Furthermore, LINC00511 enhanced T-cell acute lymphoblastic leukemia (T-ALL) progression by inducing miR-195-5p/LRRK1 axis [29]. In gastric cancer, LINC00511 promotes tumorigenesis through the regulation of the miR-625-5p/NFIX circuit [30]. Our findings, along with existing evidence, suggest that targeting LINC00511 might provide a potential therapeutic avenue for patients with breast cancer. The role of LINC00511 in mediating tumor formation and metastatic potential remains to be investigated in animal models. Our data identified several other lncRNAs that were enriched in the four lncRNA-based clusters. The functional roles of additional lncRNAs identified in the current study remain to be investigated.

## 4. Materials and Methods

### 4.1. Transcriptomic and Bioinformatics Analyses

Raw sequencing data from 360 TNBC and 88 normal tissue samples were retrieved from the Sequence Read Archive (SRA) database (https://www.ncbi.nlm.nih.gov/sra/SRP157974; accessed on 9 August 2021) using the SRA toolkit v2.9.2 [31]. The Kallisto index was constructed by creating a de Bruijn graph using reference transcriptome gencode.v33 and a k-mer length of 31. FASTQ files were then mapped and aligned to the generated gencode.v33 index using Kallisto v0.46, as described before [32,33,34]. Normalized transcripts per million (TPM) expression values were subsequently subjected to ICGS, UMAP dimensionality reduction, and hierarchical clustering, as described before [9,12]. ICGS2 identified clusters through a complex process of PageRank down-sampling, feature selection ICGS2, dimension reduction and clustering (sparse NMF, SNMF), cluster refinement (MarkerFinder algorithm), and cluster re-assignments using a support vector machine (SVM). The MarkerFinder algorithm was applied to identify defined clusters with unique transcriptomes. A volcano plot was used to illustrate the most differentially expressed genes (log2-fold change) vs. −log10 *p*-value. Expression of LINC00511 in the TCGA BECA cohort as function of pathological stage was retrieved from the GEPIA2 database (http://gepia2.cancer-pku.cn/#index; accessed on 9 August 2021) [35]. Expression of LINC00511 as a function of PAM50 classification or based on BC therapy was retrieved from The Atlas of non-coding RNA in Cancer (TANRIC) database (https://ibl.mdanderson.org/tanric/_design/basic/main.html; accessed on 9 August 2021) [36].

### 4.2. Ingenuity Pathway Analysis (IPA)

Upregulated genes in each lncRNA-based TNBC cluster compared to NT were imported into IPA software (Ingenuity Systems, Redwood City, CA, USA; www.ingenuity.com/; accessed on 9 August 2021) and were subjected to functional annotations, upstream regulator network analysis (URA), canonical, casual, and disease and function analyses. IPA predicts the functional regulatory networks from the gene expression profile and provides a significance tally according to the appropriate network for the set of target genes in the database. The *p*-value is the −log of *p* and signifies the possibility that target genes in the network are found together by chance [9,37]. A Z-score of −2.0 ≥ Z ≥ 2.0 was considered significant.

### 4.3. Protein–Protein Interaction Network Analysis

The upregulated genes in each lncRNA-based cluster (LINC00511-enriched, LINC00393-enriched, FIRRE-enriched, and normal-like) compared to NT were subjected to PPI network analysis using the STRING (STRING v10.5) database to illustrate the interacting genes/Proteins based on knowledge and predication, as described before [38].

### 4.4. Survival Analysis

Kaplan–Meier survival analysis and plotting were conducted using IBM SPSS statistics version 26 software. Patients were grouped according to lncRNA-based classification into LINC00511-Enr, LINC00393-Enr, FIRRE-Enr, and NT-like. The log-rank test was used to compare the outcomes between the expression groups. A similar analysis was carried out using mRNA-based TNBC clusters (BLIS, IM, MES, and LAR).

### 4.5. Generation of LINC00511 Knockout TNBC Models Using CRISPR-Cas9

Synthesis, construction, and purification of paired guide RNAs targeting the LINC00511 promoter were performed as we described before [12]. In brief, guide RNA (gRNA) sequences targeting LINC00511 promoter region (LINC00511_guide_1: 5′-TTCTAATACGACTCACTATAGTTCGGCCCTTATATACCAGGGTTTTAGAGCTAGA and LINC00511_guide_2: 5′-TTCTAATACGACTCACTATAGGAGACCTTCGAAAAACGACGGTTTTAGAGCTAGA) were designed using CRISPETa, as described before [39] and were synthesized using the EnGen sgRNA Synthesis Kit, S. pyogenes (NEB# E3322S). The transcribed guides were purified using a monarch RNA cleanup Kit (NEB# T2040L) and the eluted gRNA was stored at −80 °C until use. The concentrations of purified gRNA were measured using NanoDrop 2000 (Thermo Fisher Scientific, Waltham, MA, USA). Genomic screening for successful deletion of lINC00511 was carried out using LINC00511_screen_F: 5′-GGAATGCCAGCTTTGTCTGTG and LINC00511_screen_R: 5′ CACCGTGTCCCAGGTGAATC primers. Expression of LINC00511 in KO and WT cells was assessed using LINC00511_F: 5′-CAAGCTGGAGTCATCCCCC and LINC00511_R: 5′-CTAGAGGCTGAAGGACAACGG primers and qRT-PCR, while GAPDH (GAPDH_F: 5′-GGAGCGAGATCCCTCCAAAAT and GAPDH_R: 5′ GGCTGTTGTCATACTTCTCATGG were used as controls.

### 4.6. Cas9 RNP Transfection Using Electroporation and PCR-Based Genotyping of LINC00511

The preparation of the RNP complex and transfection were performed as we described before [12]. In brief, 1000 ng of each gRNA were mixed with 1.5 μL of 20 µM Cas-9 Enzyme (EnGen Spy Cas9 NLS (NEB# M0646M)) to form the RNP complex. HCC70 TNBC cells were pelleted and washed once with 1× PBS and then kept on ice until use. The formed RNP complex was pre-mixed with 20 µL of nucleofector solution (P3 Primary Cell 4D-Nucleofector kit, Lonza# V4XP-3024) and then mixed with TNBC cells. The complex mixture was immediately transferred into a nucleocuvette and electroporated using a 4D Nucleofector (Lonza, Basel, Switzerland). After electroporation, the cells were recovered and plated in a 6-well plate. Genomic PCR was then performed to confirm genotyping of LINC00511-promoter deleted cells, as described before [12].

### 4.7. Colony Forming Unit (CFU) Assay and Paclitaxel Sensitivity of LINC00511-KO and Parental TNBC Cells

Both control and LINC00511-KO cells were serially diluted and treated with the paclitaxel (PTX) drug (10 nM) for 7 days, followed by crystal violet (0.1% in 10% EtOH) staining. Images were taken and compared with the controls. Stained colonies were allowed to airdry at room temperature, and then CFUs were quantified by dissolving crystal violet in 5% SDS and measuring absorbance at 590 nm. Data are represented as mean ± S.D. from four technical replicas. Acridine orange and ethidium bromide (AO/Etbr) staining was used to determine the live and dead cells after exposure to different concentrations of Paclitaxel, as described before [12]. After treatment, the control and KO cells were stained with a dual fluorescent staining solution containing 100 μg/mL AO and 100 μg/mL EB (AO/EtBr, Sigma, St. Louis, MO, USA). The cells were washed and visualized under a Nikon Eclipse Ti fluorescence microscope. The differential uptake of AO/EtBr allowed for the identification of viable and non-viable cells.

### 4.8. Statistical Analysis

Statistical analyses and graphing were performed using Microsoft Excel 365 and GraphPad Prism 8.0 software. A two-tailed *t*-test was used for the comparative groups. *p*-values ≤ 0.05 were considered significant. IBM SPSS statistics version 26 was used for survival analysis.

## 5. Conclusions

Our data provide the first lncRNA-based classification of NBC and suggest its potential utilization to stratify TNBC patients for a more tailored treatment choice. However, the potential utilization of lncRNA-based stratification in clinical practice warrants further investigation.

## Data Availability

All data associated with this study are included in supplementary tables; otherwise, accession numbers are provided in methods.

## Supplementary Materials

The following supporting information can be downloaded at: https://www.mdpi.com/article/10.3390/ncrna8040044/s1, Figure S1: PPI network for genes upregulated in the FIRRE-Enr cluster; Figure S2: PPI network for genes upregulated in the LINC00511-Enr cluster; Figure S3: PPI network for genes upregulated in the LINC00393-Enr cluster; Figure S4: PPI network for genes upregulated in the NT-like cluster; Figure S5: Prognostic value of lncRNA-based classification; Figure S6: Effect of CRISPR-Cas9 mediated LINC00511 promoter depletion on MDA-MB-157 colony formation and sensitivity to paclitaxel; Table S1: Differentially expressed lncRNA transcripts in TNBC (n = 360) vs. controls (n = 88) title; Table S2: Iterative Clustering and Guide-gene Selection (ICGS) classification of TNBC and normal BC; Table S3: Gene enrichment in the indicated TNBC or normal breast cluster; Table S4: Enriched gene ontology (GO) terms in the FIRRE-Enr TNBC cluster based on STRING analysis; Table S5: Enriched gene ontology (GO) terms in the LINC00511-Enr TNBC cluster based on STRING analysis; Table S6: Enriched gene ontology (GO) terms in the LINC00393-Enr TNBC cluster based on STRING analysis; Table S7: Enriched gene ontology (GO) terms in the NT-like TNBC cluster based on STRING analysis.
