# Supplementary material for: LncRNA-Based Classification of Triple Negative Breast Cancer Revealed Inherent Tumor Heterogeneity and Vulnerabilities
Source: Noncoding RNA. 2022 Jun 21;8(4):44. doi: 10.3390/ncrna8040044 (PMC9326727; doi:10.3390/ncrna8040044)
Supplement: Supplementary file 1 [file ncrna-08-00044-s001.zip › Supplementary figure S4.pdf]

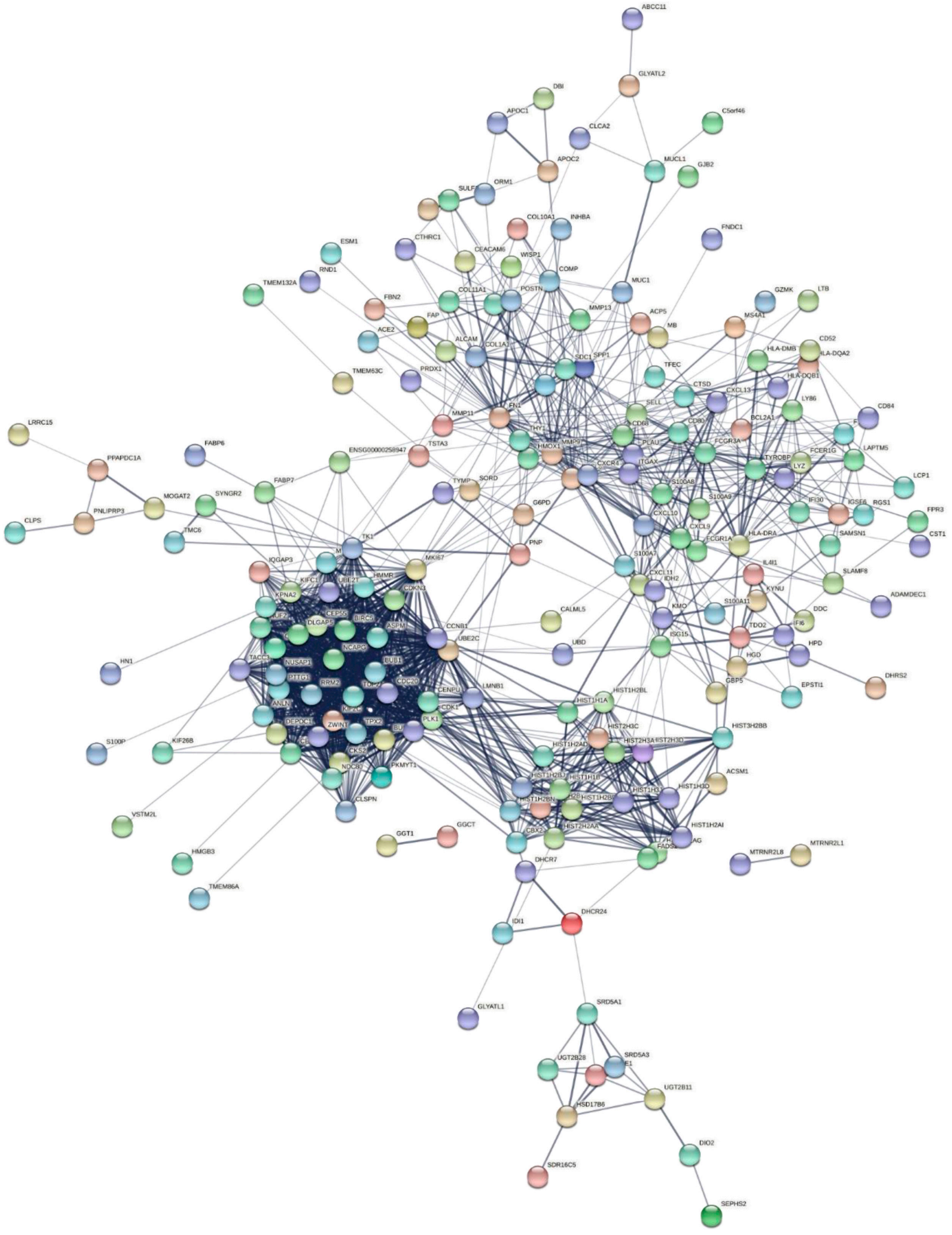

Supplementary figure S4. PPI network for genes upregulated in NT-like cluster. Network statistics: network exhibited 234 nodes, 1,428 number of edges, an average node degree of 12.2, and average local clustering coefficient of 0.569 and PPI enrichment p-value of < 1.0e-[16] .
