# Supplementary material for: LncRNA-Based Classification of Triple Negative Breast Cancer Revealed Inherent Tumor Heterogeneity and Vulnerabilities
Source: Noncoding RNA. 2022 Jun 21;8(4):44. doi: 10.3390/ncrna8040044 (PMC9326727; doi:10.3390/ncrna8040044)
Supplement: Supplementary file 1 [file ncrna-08-00044-s001.zip › Supplementary figure S5.pdf]

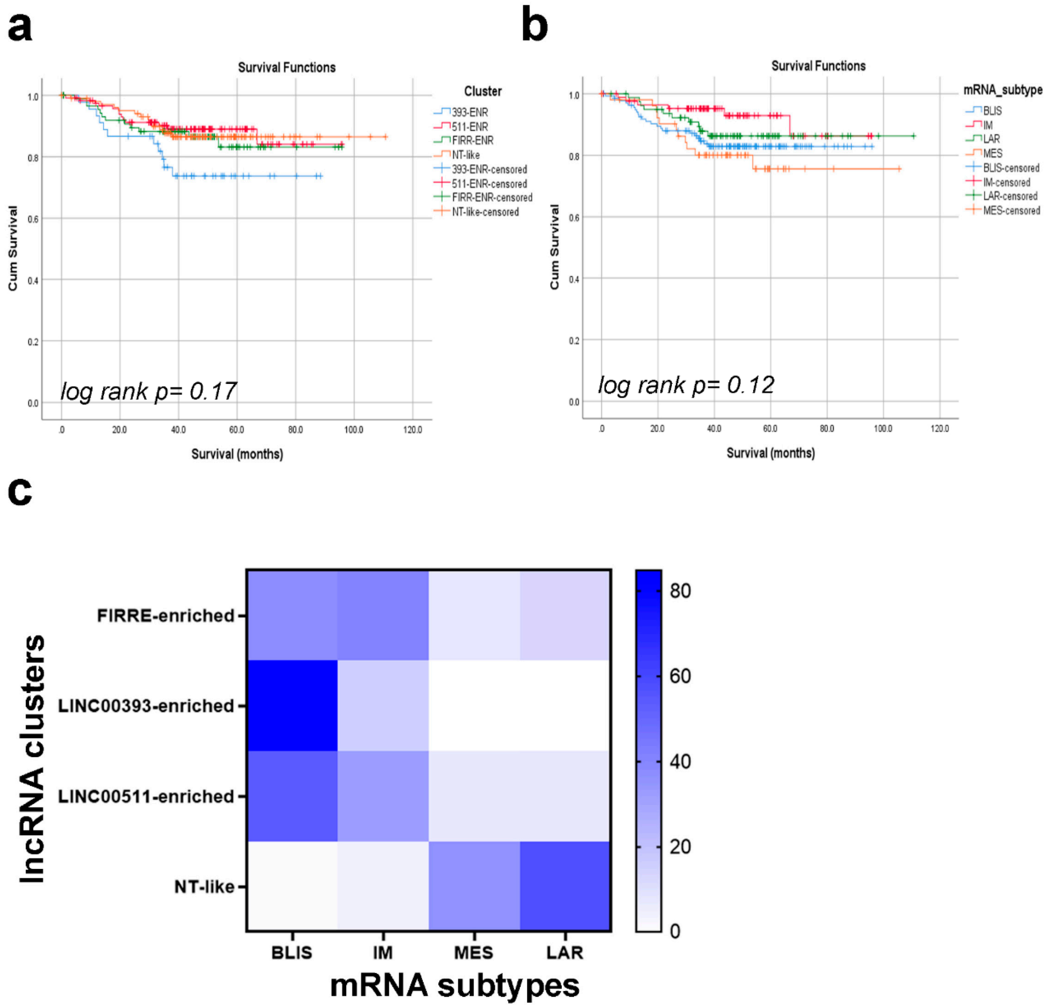

Supplementary figure S5. Prognostic value of lncRNA-based classification. (a) survival analysis of 360 TNBC patients as a function of their lncRNA-derived clusters. Log rank test was used for group comparisons. **(b)** survival analysis of same TNBC cohort as function of their mRNA-derived cluster (BLIS, IM, MES, and LAR). **(c)** Correlation matrix showing the correlation between the four lncRNA-based and mRNA-based TNBC clusters.
