# Supplementary material for: LncRNA-Based Classification of Triple Negative Breast Cancer Revealed Inherent Tumor Heterogeneity and Vulnerabilities
Source: Noncoding RNA. 2022 Jun 21;8(4):44. doi: 10.3390/ncrna8040044 (PMC9326727; doi:10.3390/ncrna8040044)
Supplement: Supplementary file 1 [file ncrna-08-00044-s001.zip › Supplementary figure S6.pdf]

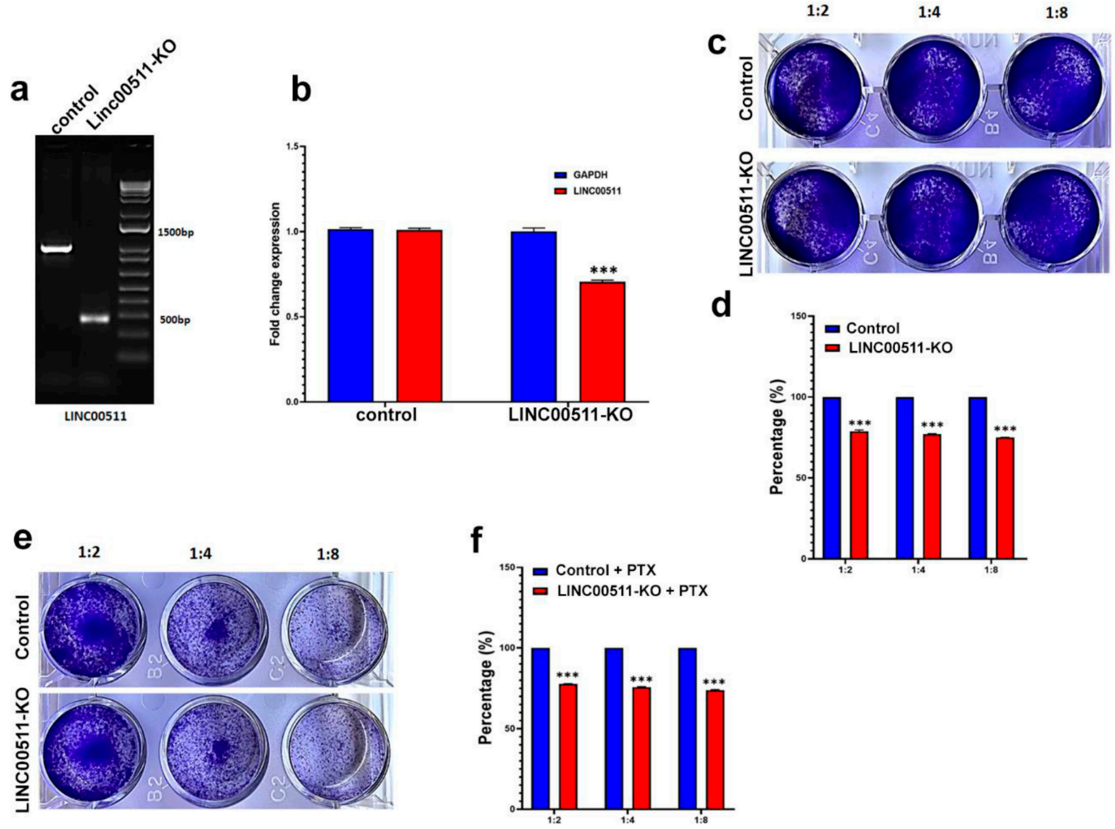

Supplementary figure S6. Effect of CRISPR-Cas9 mediated LINC00511 promoter depletion on MDA-MB- 157 colony formation and sensitivity to paclitaxel. (a) Genomic deletion of ~ 700 bp from LINC00511 promoter region using CRISPR-Cas9 in MDA-MB-157 TNBC model. qRT-PCR for LINC00511 expression in parental and LINC00511-KO MDA-MB-157 cells. Data are presented as mean ± SD, n = 3. ***p < 0.0005. Clonogenic assay for MDA-MB-157 parental and LINC00511-KO cells. **(d)** Quantification of CFU from panel c. **(d)** Clonogenic assay for MDA-MB-157 parental and LINC00511-KO cells in the presence of 10 nM Paclitaxel. **(e)** Quantification of CFU from panel d. ***p < 0.0005.
