# Supplementary material for: LncRNA-Based Classification of Triple Negative Breast Cancer Revealed Inherent Tumor Heterogeneity and Vulnerabilities
Source: Noncoding RNA. 2022 Jun 21;8(4):44. doi: 10.3390/ncrna8040044 (PMC9326727; doi:10.3390/ncrna8040044)
Supplement: Supplementary file 1 [file Supplementary figure S1.pdf › Supplementary figure S1.pdf]

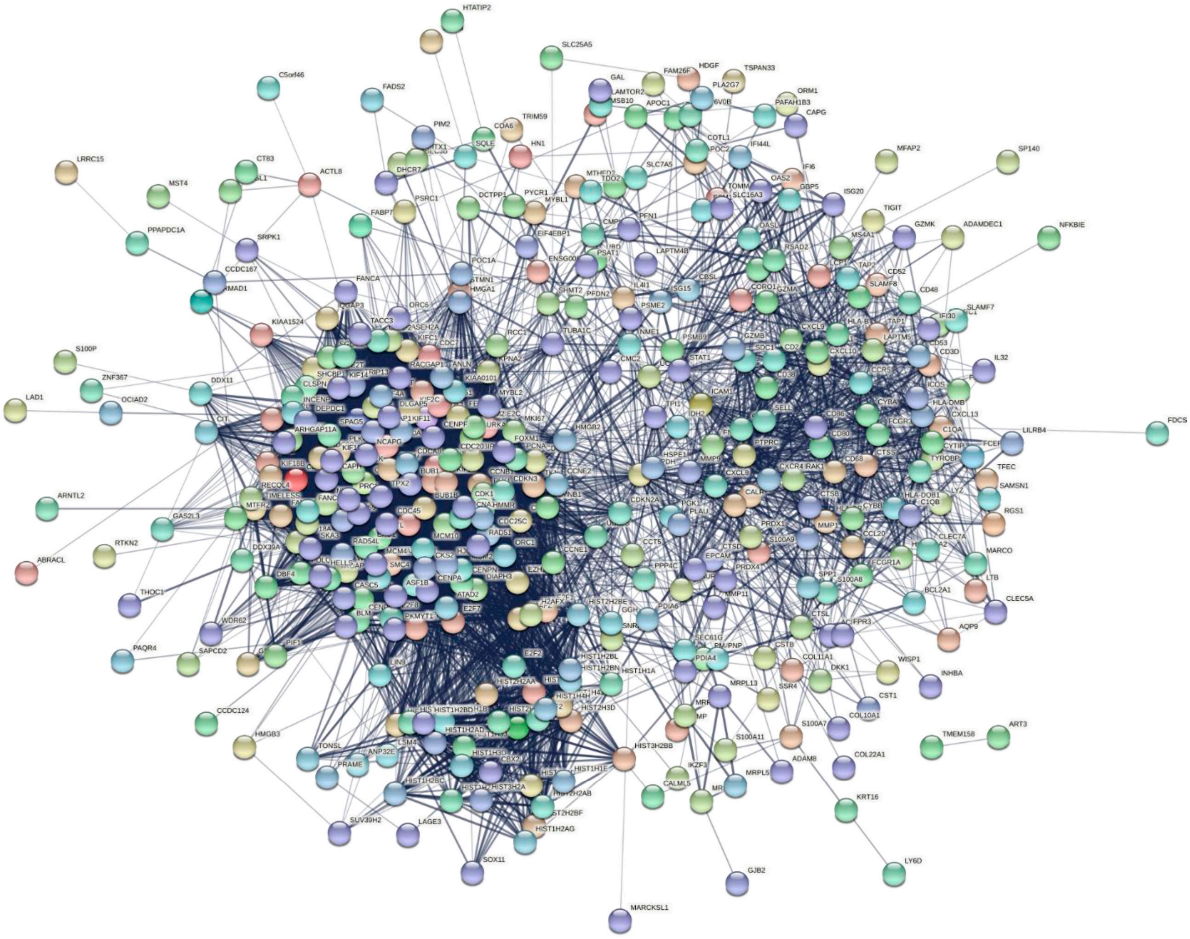

Supplementary figure S1. PPI network for genes upregulated in FIRRE -Enr cluster.

Network statistics: network exhibited 429 nodes, 9,277 edges with an average node degree of 43.2 and average local clustering coefficient of 0.568 and PPI enrichment p-value of < 1.0- 16.
