# Supplementary material for: LncRNA-Based Classification of Triple Negative Breast Cancer Revealed Inherent Tumor Heterogeneity and Vulnerabilities
Source: Noncoding RNA. 2022 Jun 21;8(4):44. doi: 10.3390/ncrna8040044 (PMC9326727; doi:10.3390/ncrna8040044)
Supplement: Supplementary file 1 [file Supplementary figure S2.pdf › Supplementary figure S2.pdf]

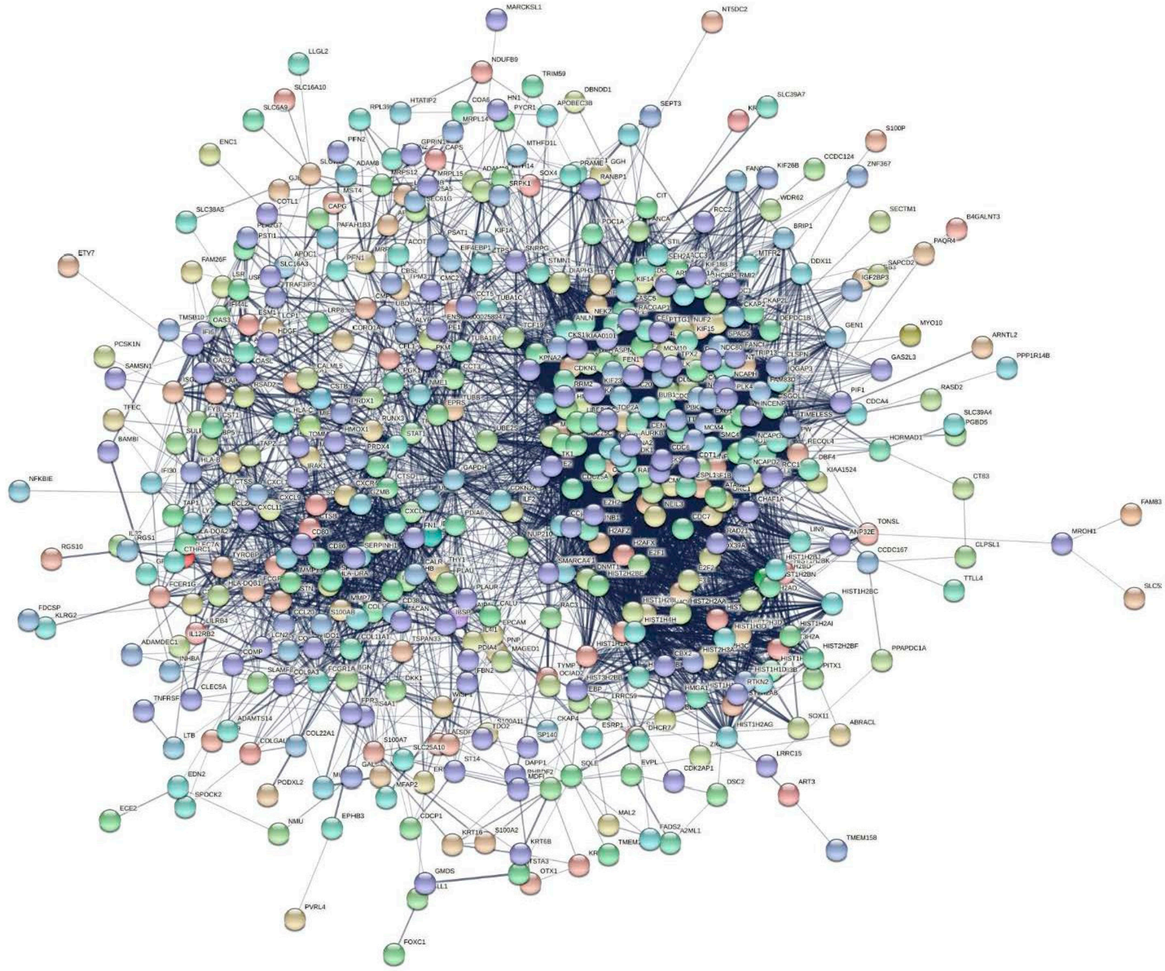

Supplementary figure S2. PPI network for genes upregulated in LINC00511-Enr cluster.

Network statistics: network exhibited 539 nodes, 11,067 edges, with an average node degree of 41.1 and avg. local clustering coefficient of 0.531 and PPI enrichment p-value of $< 1.0e^{-16}$.
