# Supplementary material for: LncRNA-Based Classification of Triple Negative Breast Cancer Revealed Inherent Tumor Heterogeneity and Vulnerabilities
Source: Noncoding RNA. 2022 Jun 21;8(4):44. doi: 10.3390/ncrna8040044 (PMC9326727; doi:10.3390/ncrna8040044)
Supplement: Supplementary file 1 [file Supplementary figure S3.pdf › Supplementary figure S3.pdf]

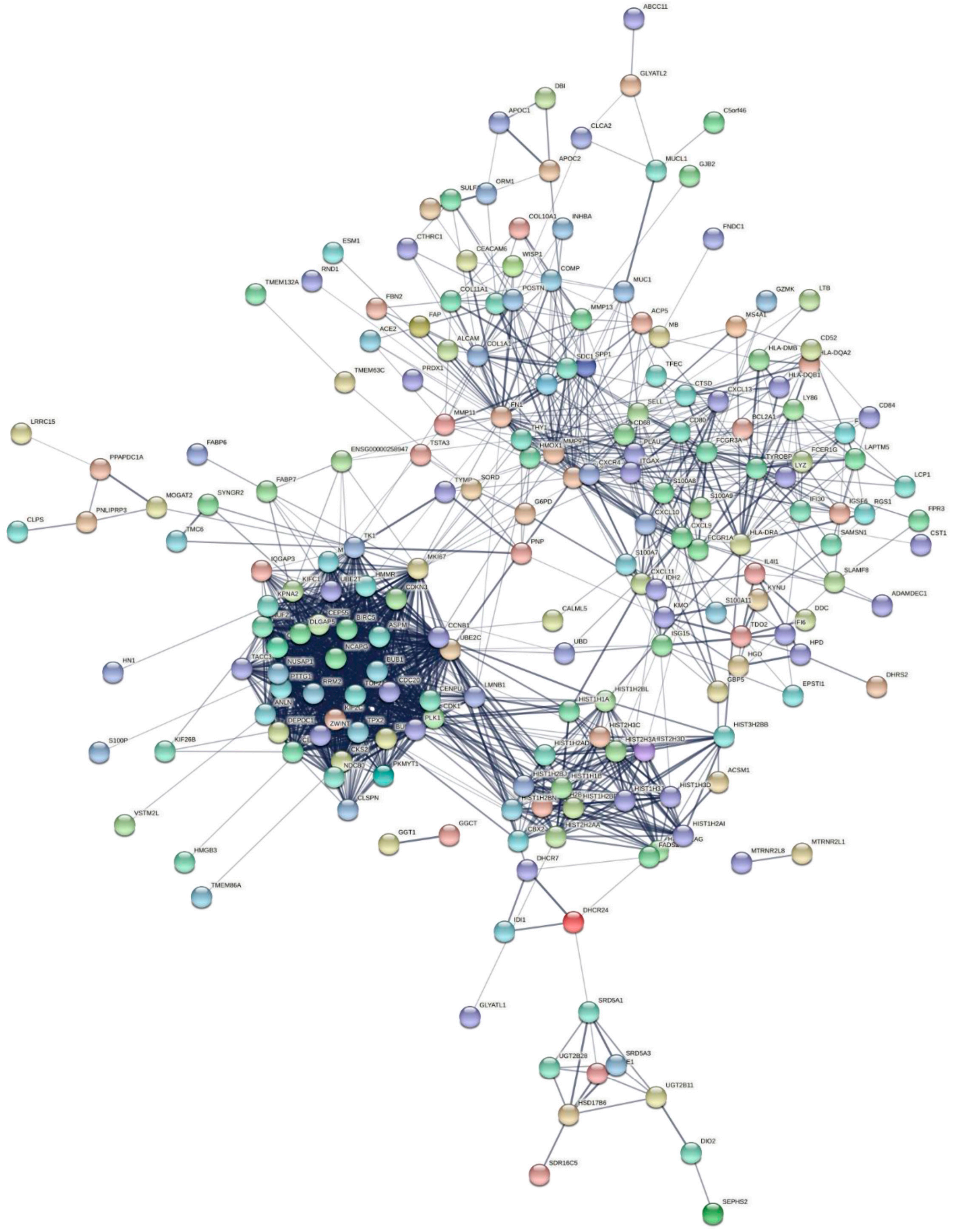

Supplementary figure S3. PPI network for genes upregulated in LINC00393-Enr cluster.

Network statistics: Network exhibited 492 nodes, 9799 edges, an average node degree of 39.8, and avg. local clustering coefficient of 0.538. The network exhibited PPI enrichment p-value of < $1.0e^{-16}$.
